# Cadaveric and CT angiography study of vessels around the transverse colon mesentery

**DOI:** 10.1186/s12957-023-02919-9

**Published:** 2023-02-06

**Authors:** Yusuke Ogi, Hiroyuki Egi, Kei Ishimaru, Shigehiro Koga, Motohira Yoshida, Satoshi Kikuchi, Satoshi Akita, Hiroki Sugishita, Hironori Matsumoto, Tetsuya Shimokawa, Akihide Takeuchi, Yuji Watanabe

**Affiliations:** 1 Division of Gastrointestinal Surgery and Surgical Oncology, Ehime University Hospital, Toon City, Ehime 454 Shitsukawa791-0295 Japan; 2 Division of Developmental Biology and Functional Genomics, Graduate School of Medicine, Ehime University, 454 Shitsukawa, Toon City, Ehime 795-0295 Japan

**Keywords:** Middle colic artery, Accessory middle colic artery, Anatomy, Transverse colon, Inferior mesenteric vein

## Abstract

**Background:**

Laparoscopic and robotic surgery for transverse colon cancer are difficult due to complex fusion of the foregut and midgut and variation of the vessels of the transverse colon. Although the vessels of the right colon have been investigated, middle colic artery (MCA) variation and the relationship with vessels around the transvers colon are unknown. We investigated variation of the MCA using computed tomography angiography (CTA) and cadaver specimen and the relationship between the superior mesenteric vein (SMV) and MCA using CTA. The classification of vessels around the transverse colon may lead to safer and reliable surgery.

**Methods:**

This study included 505 consecutive patients who underwent CTA in our institution from 2014 to 2020 and 44 cadaver specimens. Vascular anatomical classifications and relationships were analyzed using CT images.

**Results:**

The MCA was defined as the arteries arising from the superior mesenteric artery (SMA) that flowed into the transverse colon at the distal ends. The classifications were as follows: type I, branching right and left from common trunk; type II, the right and left branches bifurcated separately from the SMA; and type III, the MCA branched from a vessel other than the SMA. Type II was subclassified into two subtypes, type IIa with one left branch and type IIb with two or more left branches from SMA. In the CTA and cadaver studies, respectively, the classifications were as follows: type I, *n* = 290 and *n* = 31; type IIa, *n* = 211 and *n* = 13; type IIb, *n* = 3 and *n* = 0; and type III, *n* = 1 and *n* = 0. We classified the relationship between the MCA and left side of the SMV into three types: type A, a common trunk runs along the left edge of the SMV (*n* = 173; 59.7%); type B, a right branch of the MCA runs along the left edge of the SMV (*n* = 116; 40.0%); and type C, the MCA runs dorsal of the SMV (*n* = 1; 0.3%).

**Conclusions:**

This study revealed that The MCA branching classifications and relationship between the SMV and MCA. Preoperative CT angiography may be able to reliably identify vessel variation, which may be useful in clinical practice.

## Background

Colorectal cancer is increasing worldwide. In Japan, the number of morbidities is also increasing. The standard strategy for colorectal cancer is surgical resection. Laparoscopic and robotic surgery have become widespread as minimally invasive approaches for colorectal cancer. Clinical studies showing the benefits of laparoscopic and robotic surgery often excluded transverse colon cancer [1–3]. Laparoscopic and robotic surgery for transverse colon cancer are associated with a greater degree of difficulty because of the complex fusion of the foregut and midgut and variation of the vessels of the transverse colon.

The frequency of ileocolic artery and right colic artery and vein variations [4–12] and accessary middle colic artery [13–16] has been reported, but few of studies have focused on the middle colic artery (MCA) [17, 18], and few studies have focused on the relationship between the MCA and superior mesenteric vein (SMV) and demonstrated vessel abnormalities using preoperative and perioperative visualization [19–21]. We considered that the relationship between the MCA and SMV has been very important in the case of lymph node dissection around the MCA. We considered that the safety and accuracy of lymph node dissection in transverse colon cancer have been increased based on research on vessels around the transverse colon mesentery.

We researched the variation of MCA bifurcation using cadaver specimens and dynamic computed tomography angiography. Moreover, using dynamic computed tomography (CT), we researched the relationship between the MCA and SMV and the frequency of variation of the MCA and artery of Moskowitz and the confluence of the inferior mesenteric vein (IMV).

In this study, we investigated the vessel variation and the interrelationships among the arteries and veins related to blood supply to the transverse colon. We considered that the classification of the variation and the frequency of the variation would be practically useful.

## Methods

### Patients

In the present study, 505 consecutive patients (male, *n* = 329; female, *n* = 176; median age 70 years [range, 21–97 years]) who underwent CT angiography at Ehime University Hospital from January 2014 to December 2020 were prospectively enrolled.

### Cadavers

A total of 44 cadavers were dissected (male, *n* = 18; female, *n* = 26). The cadavers were excluded if there was any evidence of previous upper abdominal surgery or if peritoneal dissemination of digestive cancer was observed macroscopically in the peritoneal cavity.

### CT angiography protocol and workstation

Patients underwent three-dimensional (3D) CT angiography using a 96- or 128- or 320-detector CT scanner. The tube potential was 120 kVp, and the tube current was adjusted by automatic exposure control with a noise index of 10 and a slice thickness of 0.5 mm. Iomeprol (350 mg I/ml, iomeron 350; Eisai, Tokyo, Japan) was used as contrast agent. Patients were injected with 0.6 g/kg iodine (upper limit, 47.25g) for 30 s at a rate of 2.6–4.5 ml/s. The bolus tracking method was initiated when the contrast in the thoracic aorta (diaphragm level) reached 150 Hounsfield units. Sixty seconds after the injection of iomeprol, the venous phase image was acquired. One hundred twenty seconds after the injection of contrast agent, the equilibrium phase image was acquired. An image processing analysis was performed using a 3D volume rendering technique with the Advantage Workstation system version 4.5 (GE Health care, Tokyo, Japan).

### Definition of the middle colic artery and accessory middle colic artery

The MCA was defined as the arteries arising from the SMA that flowed into the transverse colon at the distal ends. In this study, when the MCA had two or three branches, the distance from the caudal branch was measured. The branching from vessels other than the SMA into the transverse colon was defined as the accessory middle colic artery. These definitions were developed according to 3D-CT images and axial and coronal CT scans. When the right and left branch branched from the common trunk, the length of the common trunk from the SMA was measured.

In the cadavers, the SMA was identified and the ventral peritoneum was incised. Then the SMA was dissected ventrally to reach the branch of the MCA. The first branch was dissected to the distal end, the right and left branches were identified, and the length of the common trunk was measured. In addition, the ventral dissection of the SMA was extended on the cranial side to check for other branches.

### Intersectional patterns of the MCA and SMV

The MCA has a right branch that feeds the right side of the transverse colon and a left branch that feeds the left side. We evaluated the vessels running left ventral side of the SMV. We classified the MCA as common trunk running left on the ventral side of the SMV and a right branch; when there were two or more branches of the MCA, the first branch from the SMA was considered to be the right branch.

### The artery of Moskowitz

The Moskowitz artery was defined as the vessel that directly anastomoses the middle and left colic artery. The Moskowitz artery was detected using 3D- and coronal two-dimensional CT images.

### Drainage site of the inferior mesenteric vein (IMV)

The major portal vein is formed by the confluence of the splenic vein, SMV, and IMV. The IMV confluence was detected according to axial and coronal CT images in the venous phase.

### Statistical analyses

All statistical analyses were performed using EZR (Saitama Medical Center, Jichi Medical University, Saitama, Japan), which is graphical user interface for R (The R Foundation for Statistical Computing, Vienna, Austria). More precisely, it is a modified version of R commander designed to add statistical functions frequently used in biostatistics [22].

The results are expressed as the number of cases evaluated. Fisher’s exact test was used for categorical variables, and the Mann-Whitney *U* test was used for continuous variables. All *P* values were two-tailed. *P* values of < 0.05 were considered to indicate statistical significance.

## Results

### Classification of the MCA bifurcation

**Fig. 1:**
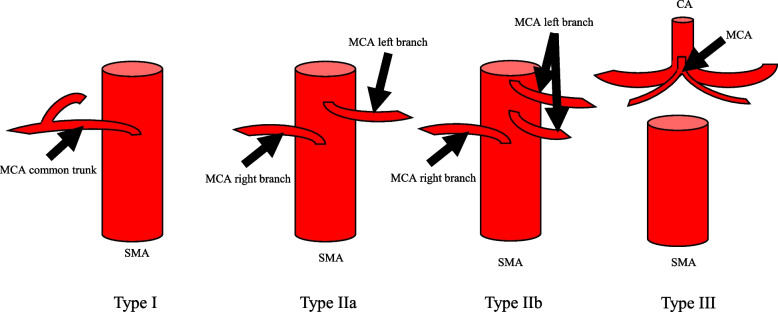
Classification of the MCA bifurcation. Type I, one right branch and one left branch of the MCA branch from the common trunk. Type II, one right branch and one left branch of the MCA branch separately from the SMA. Type II is divided into two subtypes. Type IIa, one right branch and one left branch of the MCA branch from the SMA. Type IIb, one right branch and two left branches of the MCA branch from the SMA. Type III, an MCA branches from a vessel other than the SMA. CA, celiac artery; MCA, middle colic artery; SMA, superior mesenteric artery

First, we classified middle colic arterial branching variation into three types (Fig. 1). According to the CT angiography and cadaver studies, respectively, the variation was classified as follows: type I, *n* = 290 and *n* = 31; type IIa, *n* = 211 and *n* = 13; type IIb, *n* = 3 and *n* = 0; and type III, *n* = 1 and *n* = 0 (Table 1). The frequency in the CT angiography and cadaver studies did not differ to a statistically significant extent (*p* = 0.182).

**Table 1:** The classification of MCA branch pattern

Features	CT angiography	Cadaver
Total number	505	44
The type of MCA bifurcation, *n* (%)		
Type I	290 (57.4%)	31 (70.5%)
Type IIa	211 (41.8%)	13 (29.5%)
Type IIb	3 (0.6%)	0
Type III	1 (0.2%)	0

### The length of the common trunk of the MCA

We investigated the length of the common trunk from the SMA to the bifurcation in type I. The length in the CT angiography and cadaver examinations was 2.6 cm (median, 0.1–9.0) and 2.8 cm (median, 0.5–6.3), respectively. There was no significant difference between the CT and cadaver studies (*p* = 0.713).

### The distance from the first branch to other branches

In type II, the MCA has one or more branches from the SMA. Type IIa has one other branch and type IIb has two or more branches. In type IIa, the median distance was 1.8 cm (0.2–4.7 cm).

### The relationship between the SMV and MCA

**Fig. 2:**
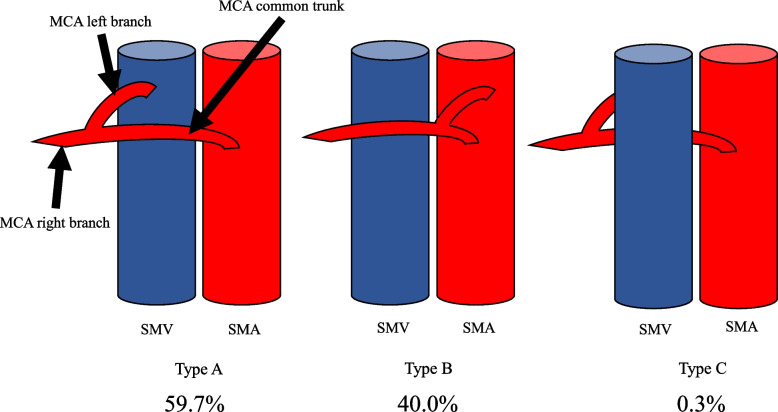
The relationship between the SMV and the MCA. Type A, a common trunk runs along the left edge of the SMV. Type B, the right branch of the MCA runs along the left edge of the SMV. Type C, the MCA runs dorsal of the SMV. MCA, middle colic artery; SMV, superior mesenteric vein

We classified the relationship between the MCA and the left side of the SMV into three types: in type A, a common trunk runs along the left edge of SMV (*n* = 173; 59.7%); in type B, a right branch of the MCA runs along the left edge of the SMV (*n* = 116; 40.0%); and in type C, the MCA runs dorsal of the SMV (*n* = 1; 0.3%) (Fig. 2).

### Origin of the accessory MCA and the artery of Moskowitz

Eighteen cases had an accessory MCA. In SMA flow, 3 cases had a jejunal artery and 6 cases had a pancreatic artery. In celiac flow, 1 case had a celiac artery and 1 case had a common hepatic artery and 7 cases had a splenic artery (Table 2). A Moskowitz artery was present in 12 cases (2.4%).

**Table 2:** Origin of the AMCA

Features	Value
Total number	18
Origin, *n* (%)	
SMA flow	
Jejunal artery	3 (16.7%)
Pancreatic artery	6 (33.3%)
Celiac flow	
Celiac artery	1 (5.5%)
Common hepatic artery	1 (5.5%)
Splenic artery	7 (38.9%)

### Drainage site of IMV

**Fig. 3:**
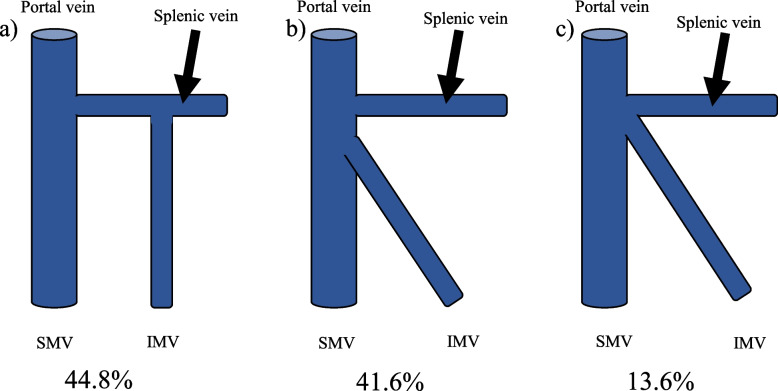
The drainage site of the inferior mesenteric vein (IMV). **a** The IMV flows into the splenic vein and forms the portal vein. **b** The IMV flows into the superior mesenteric vein and forms the portal vein. **c** The IMV, SMV and splenic vein merge simultaneously to form the portal vein. IMV, inferior mesenteric vein; SMV, superior mesenteric vein

We classified the drainage site of the IMV into three types: splenic vein (*n* = 226; 44.8%); SMV (*n* = 210; 41.6%); and simultaneous merging (*n* = 69; 13.6%) (Fig. 3).

## Discussion

The present study demonstrated three main points. The first point was the middle colic artery (MCA) branchial variation classification and frequency of accessory MCA (AMCA) branching from vessels other than the superior mesenteric artery (SMA) in cadaver and CT angiography studies. The second point was the relationship between the left edge of the superior mesenteric vein (SMV) and the MCA branch. The third point was the frequency of inferior mesenteric vein (IMV) drainage site.

**Fig. 4:**
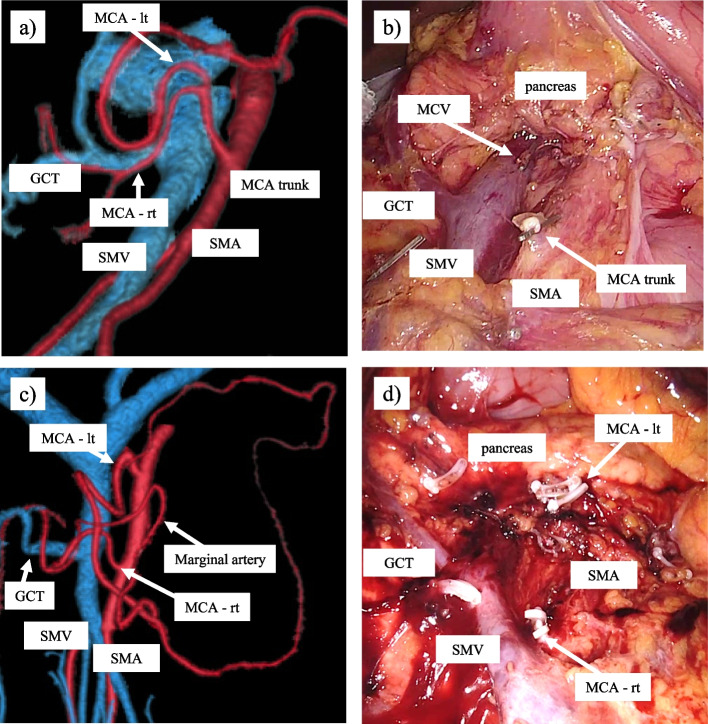
CT angiography and intraoperative findings of type I and type IIa MCA bifurcation. **a** CT imaging of a type I MCA bifurcation. The right and left branches of the MCA branch from the common trunk. **b** Intraoperative findings after main lymph node dissection by cutting the common trunk for extended right hemicolectomy in a case with a type I MCA. **c** CT imaging of a type IIa MCA bifurcation. The right and left branches of the MCA branch separately from the SMA. **d** Intraoperative view after main lymph node dissection with ligation of the right and left branches of a type IIa MCA for extended right hemicolectomy. CT, computed tomography; MCA, middle colic artery; MCV, middle colic vein; SMV, superior mesenteric vein; GCT, gastrocolic trunk

Regarding the middle colic artery branchial variation classification and frequency of AMCA branching from vessels other than the SMA in cadaver and CT angiography studies, many reports have described the anatomy of the ileocolic and right colic artery and vein [4–12, 23–25]. Moreover, the effect of lymph node dissection in the right colectomy has been reported [26, 27]. Surgery for transverse colon cancer is highly difficult because of the many variations of the MCA and SMV branches. Clinical trials that provided evidence related to laparoscopic surgery, excluded subjects with transverse colon cancer due to the high degree of difficulty in these operations [1–3, 28, 29]. Since then, an approach to safely perform surgery for transverse colon cancer has been reported [30] and has gradually become popular. Hence, an understanding of the anatomy of the branching of the MCA is important for lymph node dissection. This current study presented the variation of these branch types and the frequency of the AMCA. According to the tumor location, lymph node dissection along the MCA is required for adequate dissection [31–33]. However, by cutting the root of the MCA, residual intestinal blood flow may be reduced, and the rates of postoperative complication such as anastomotic leakage may be higher. In the case of colon cancer surgery involving the liver flexure, while the main lymph node is dissected by cutting at the middle colic trunk in type I cases, the main lymph nodes are resected by isolating the right branch of the MCA in type II cases, and the residual transverse flow can be sufficient for the left branch flow. However, for middle or left transverse colon cancer, in type II, both branches of the MCA must be ligated in order to perform central vascular ligation (Fig. 4). In a CT angiography study, some cases were reported in which there was no margin at Griffiths’ point [34]; this was disadvantageous as peripheral vessels were not adequately contrasted [35]. The studies comparing preoperative CT angiography and intraoperative findings had shown that CT angiography was able to accurately detect the vessel root of the mesentery [19, 20]. On the other hand, cadaver research for colon vessels has been reported. In the cadaver study, in all cases, the splenic flexure was supplied by the marginal artery, and there was no area with poor blood flow [36]. Improvements in computed tomography quality have enabled the vessel construction to be studied in detail.

The analysis of the frequency of the AMCA has been reported [13–16]. Some studies focused on the splenic flexure and its relationship to the left colic artery [37, 38]. The analytical methods have been reported, with some studies using CT angiography and others using cadavers; the AMCA was reported to be present in 6.8–49.2%. In the present study, the MCA was defined as branching from the SMA. A recent meta-analysis reported the frequency of the AMCA and AMCA at the root. In the meta-analysis, an MCA was defined as an AMCA in a case in which it had a branch away from the first MCA branch [4]. Based on the results of our study, it is possible to reclassify vessels classified as an AMCA based on the proximal distance from the first MCA branch.

In the present study, the Moskowitz artery [39–41] was found in 2.4% of patients. The marginal artery was present in all cadavers in our cadaver study. We considered that the Moskowitz artery may develop laterally in collateral circulation due to stenosis or occlusion of the IMA. In our cases in which a Moskowitz artery was found, the calcification around the IMA was stronger in comparison to other cases.

The second point was the relationship between the left edge of the SMV and the MCA branch. It was considered that the SMV could be used as a landmark for lymph node dissection in the region of the MCA. The classification of the relationship between MCA bifurcation position and left edge of SMV has been reported [21]. In this study, we showed the patterns of intersection between the left edge of the SMV and the MCA branch and the frequency of each relationship [42]. It was suggested that the SMV could be used a landmark for sufficient and necessary lymph node dissection.

The third point was revealing the frequency of the IMV drainage site. The complex fusion of the fascial structures of the foregut, midgut, and hindgut makes resection for transverse colon cancer more difficult. Although the frequency of arterial branch variations has been investigated, confluence of the IMV had been reported to be very rare. It has been accepted that the IMV merges with the splenic vein. However, cadaver studies have reported that in this confluence, the IMV drainages to the splenic vein or SMV or the simultaneous SMV, IMV, and splenic vein [43]. Moreover, it has been reported—based on CT angiography and a physical model study—that the IMV drains to the splenic vein, the SMV, or the jejunal vein [44]. In our study, we revealed the frequency of IMV merging using CT angiography and classified the types of confluence. This result supported the theory that the IMV and portal vein are the main flow vessels during embryonic development. It was considered that the diameter of the IMV was relatively narrower because of the abundant blood flow in the SMV and splenic vein.

The present study was associated with some limitations. First, vascular bifurcation was detected on CT angiography but was not confirmed during actual surgery. However, we considered that CT angiography was useful because of reports comparing it to actual intraoperative findings. Second, the definitions of the MCA and AMCA differed in each study. In our study, we considered that the MCA branched from the SMA and showed the distance between each branch and the length of the common trunk. Third, the present study was conducted in a single institution and only analyzed Japanese subjects.

## Conclusions

We reported our MCA branching classifications and the relationship between the left edge of the SMV and the MCA. We considered that preoperative CT angiography may be able to reliably identify vessel variations, which may be practically useful.

## Data Availability

Not applicable.

## Abbreviations

MCA: Middle colic artery
SMV: Superior mesenteric vein
CT: Computed tomography
IMV: Inferior mesenteric vein
3D: Three dimensional
AMCA: Accessory middle colic artery
GCT: Gastrocolic trunk
